# Report of the Medical Records of the Mutual Life Insurance of New York

**Published:** 1901-02

**Authors:** 


Report of the Medical Records of the Mutual Life Insurance of New York. For fifty-six years – 1843 to 1898. By Elias J. Marsh, M. D., and Granville M. White, M. D., Medical Directors. Published by the Company. 1900.

This very interesting report was prepared by the medical directors of the Mutual Life Insurance Company of New York, to accompany the diagrams and tables exhibited by the company at the recent Paris exhibition, representing the mortality records of that company from the commencement of its business in 1843 to the end of the year 1898. The tables are accompanied by colored diagrams, in which each disease has some distinctive color in each age period, showing at a glance the comparative mortality with other diseases. These tables are most ingeniously constructed and show an immense amount of labor and skill to prepare them. The tables showing the mortality percentage of the individual diseases at each period of life, also deserve great praise, and all testify to the careful collection and arrangement of facts and figures made by the medical officers of the company.

Some interesting facts about these tables may not be amiss. During the fifty-six years of existence, there have been in all 46,525 deaths. In the year 1884 there were only three deaths; in the year 1898, there were 3,421 deaths. These large figures for 1898 show partly to what a monumental size the company has grown and the beneficence bestowed by it upon thousands in their hour of greatest need.

The company is, indeed, to be congratulated in having at the head of its medical board two such capable and efficient men as Drs. Marsh and White and these tables show what good use can be made of the facts gathered by the large insurance companies.

The book is handsomely prepared and published in the company's own printery. W. C. K.

